# A phased intervention bundle to decrease the mortality of patients with extracorporeal membrane oxygenation in intensive care unit

**DOI:** 10.3389/fmed.2022.1005162

**Published:** 2022-10-17

**Authors:** Yajun Jing, Zhiyong Yuan, Weigui Zhou, Xiaoning Han, Qi Qi, Kai Song, Jinyan Xing

**Affiliations:** ^1^Department of Critical Care Medicine, The Affiliated Hospital of Qingdao University, Qingdao University, Qingdao, China; ^2^School of Mathematics and Statistics, Qingdao University, Qingdao, China

**Keywords:** ECMO, intervention bundle, compliance, mortality, complication

## Abstract

**Aim:**

To evaluate whether a phased multidimensional intervention bundle would decrease the mortality of patients with extracorporeal membrane oxygenation (ECMO) and the complication incidence.

**Materials and methods:**

We conducted a prospective observational study in comparison with a retrospective control group in six intensive care units (ICUs) in China. Patients older than 18 years supported with ECMO between March 2018 to March 2022 were included in the study. A phased intervention bundle to improve the outcome of patients with ECMO was developed and implemented. Multivariable logistic regression modeling was used to compare the mortality of patients with ECMO and the complication incidence before, during, and up to 18 months after implementation of the intervention bundle.

**Results:**

The cohort included 297 patients in 6 ICUs, mostly VA ECMO (68.7%) with a median (25th–75th percentile) duration in ECMO of 9.0 (4.0–15.0) days. The mean (SD) APECHII score was 24.1 (7.5). Overall, the mortality of ECMO decreased from 57.1% at baseline to 21.8% at 13–18 months after implementation of the study intervention (*P* < 0.001). In multivariable analysis, even after excluding the confounding factors, such as age, APECHII score, pre-ECMO lactate, and incidence of CRRT during ECMO, the intervention bundle still can decrease the mortality independently, which also remained true in the statistical analysis of V-V and V-A ECMO separately. Among all the ECMO-related complications, the incidence of bloodstream infection and bleeding decreased significantly at 13–18 months after implementation compared with the baseline. The CUSUM analysis revealed a typical learning curve with a point of inflection during the implementation of the bundle.

**Conclusion:**

A phased multidimensional intervention bundle resulted in a large and sustained reduction in the mortality of ECMO that was maintained throughout the 18-month study period.

**Clinical trial registration:**

[ClinicalTrials.gov], identifier [NCT05024786].

## Introduction

The applications of extracorporeal membrane oxygenation (ECMO) for critical illness have been growing exponentially over the last decade, mostly driven by improvement in ECMO technology and increased experience with ECMO (1, 2).

Despite favorable results, the mortality of patients receiving ECMO remains high (3, 4). Not only the urgency and complexity of ECMO operation-, but also the difficulty in the management of the primary critical illness contributes to the high mortality (5). Complication rates with ECMO are high. This is true during both cannulation and ongoing management (6). Complications include hemorrhage, stroke, limb ischemia, thrombosis, and infection from the indwelling lines/tubes. Data show that at least one significant complication occurs in over half of patients during ECMO (7). Suddenly occurred mechanical-related complications called for immediate intervention and might cause catastrophic consequences (5). The initiation of ECMO is time critical and so is the ongoing management as any issue during the therapy can irreversibly compromise the patient outcome (8). A series of preventive interventions have to be developed and implemented during the ECMO procedure to avoid these potentially life-threatening affairs and to be sure that the patient is maximally supported once committed to the ECMO support.

A multidimensional intervention bundle aimed at ECMO operation and management has been developed based on previous literature (9–12) and the experience of our centers. The objective of the study was to evaluate the effect of the intervention up to 18 months after its implementation.

## Materials and methods

### Study design and population

Six adult medical and surgical Intensive Care Units (ICUs) in China were chosen as the study centers. Patients over 18 years old who received ECMO support in the ICUs between March 2018 and March 2022 were included, excluding the patients waiting for transplantation with ECMO.

We established a multidimensional intervention bundle aimed at improving the outcome of patients with ECMO through literature review and the prospective analysis of the data and the quality conferences in the six ICUs. This study was divided into the pre-intervention phase (between March 2018 and December 2019), during-intervention phase (between January 2020 and September 2020), and post-intervention phase (between October 2020 and March 2022). To coincide with the implementation periods for the study intervention, monthly data were aggregated into 6-month periods in the post-intervention phase.

Demographics, acute physiology, and chronic health evaluation (APACHE II) scores, survival after VA-ECMO (SAVE) scores and respiratory ECMO survival prediction (RESP) scores, indication for ECMO, mode of ECMO, pre-ECMO lactate, lengths of stay in ICU, days on ECMO, the outcome, and related complications were recorded.

The study was approved by the Qingdao University Affiliated Hospital Human Research Ethics Committee (QYFYKYLL931311921) and conducted according to the principles outlined by the Declaration of Helsinki. The study was registered on ClinicTrial (NCT05024786) and no consent was needed because of the observational nature of the study.

### Indication of extracorporeal membrane oxygenation

**I. V-A ECMO**: (1) cardiac arrest; (2) cardiogenic shock; (3) refractory ventricular tachycardia; (4) RV failure during LVAD support (13).

**II. V-V ECMO:** V-V ECMO should be considered in patients with severe, acute, reversible respiratory failure that are refractory to optimal medical management. The physiologic rationale for use of VV ECMO includes: (a) increasing systemic oxygenation and CO_2_ removal (ventilation), and (b) avoiding the need for injurious mechanical ventilation (14).

### Intervention bundle

We established the intervention bundle through a literature review and prospective analysis of the data and quality conferences in the centers. With the updating of related literature and our experience, new interventions were constantly being implemented. The total intervention bundle was divided into three stages.

**Interventions in the first stage** (January 2020–March 2020)—the key technology of ECMO:

**I. Education** We organized skills training including ECMO cannulation and transport for medical staff, using a variety of approaches, such as didactic lectures, skills stations, and immersive scenario-based simulation (15). Journal club was conducted weekly; each ECMO case was reviewed and summarized, and quality control meetings were held monthly to summarize experience and lessons.

**II. Supervision of aseptic operations** The awareness of aseptic manipulation was reinforced during every shift handover. We designated an infection control practitioner for every shift to supervise the whole operation process and to stop the procedure, except in an emergency, if they observed a violation of aseptic operations. All the catheters have to be fixed appropriately and the puncture point was maintained daily.

**III. Bedside ultrasound training** Ultrasound plays a key role in the safe delivery of ECMO. It not only guides the initiation of ECMO but also helps to detect and prevent some of the complications associated with ECMO. The main disadvantage of ultrasound is that it is operator dependent. Hence, appropriately trained personnel must use it to guide the management of patients on ECMO (16). Every member of the ECMO team must be certified in critical ultrasound training. In addition, we continue to strengthen the training in daily work, including the assessment of inferior vena cava, cardiac function (including systolic/diastolic and valve function), pulmonary ultrasound, cerebral blood perfusion, and other important organ function, as well as percutaneous arteriovenous puncture (17).

**Interventions in the second stage** (April 2020–July 2020)—the comprehensive management of the critical illness supported by ECMO:

**I. Ventilation strategy** Whether the patient is on either V-V or V-A mode, the ventilator should be managed at low settings to allow lung rest (18). A ventilator management protocol was developed to minimize lung injury (19), which included maintaining a low tidal volume of 4–6 ml/kg, optimum high-positive end-expiratory pressure (PEEP), and a low respiratory rate (between 8 and 10 breaths/min). The inspired fraction of oxygen (FiO_2_) through the ventilator can be minimized to reduce oxygen toxicity. Oxygenation targets are PO_2_ of greater than 60 mm Hg and O_2_ saturation of greater than 85%.

**II. Hemodynamic management** The ultimate goal of VAECMO is to reduce cardiac load and ensure tissue perfusion, mean arterial pressure (MAP), and ECMO blood flow should be titrated based on tissue perfusion (20).

(A) Tissue perfusion evaluation scheme: Physical (e.g., urine output), point-of-care ultrasound (e.g., cerebral/kidney blood flow), and laboratory (e.g., lactate) parameters. (B) All inotropes should be discontinued as soon as the VA ECMO is started, to decrease the myocardial work and allow the VA ECMO to maintain circulation. (C) If the aortic valve cannot be opened due to heart failure or the excessive left ventricle (LV) afterload caused by ECMO, a small dose of inotropes should be adopted to promote cardiac contraction and keep the aortic valve open to prevent the formation of intracardiac thrombosis or severe pulmonary edema. An intraaortic balloon pump (IABP) will be adopted to unload the LV, if necessary.

Systemic and organ perfusion were chosen as the target of hemodynamics. Systemic perfusion (above 70%) is measured by mixed venous blood saturation. Organ perfusion is measured by urine output or kidney and cerebral blood flow.

**III. Early enteral nutrition (EN)** We established a caloric goal of 25 kCal/kg/day. EN was initiated as continuous intragastric feeding at 20 mL/h *via* nasogastric tube or nasojejunal tube if not clearly contraindicated. EN rate of delivery was increased stepwise every 24 h to the target rate. This target should be reached over a period of 3 days. EN-related gastrointestinal complications were managed according to a previously published protocol (21).

**IV. Anticoagulation protocol** (22) According to the retrospective study of our center, APTT has a better correlation with heparin dosage, which can better guide heparin anticoagulation in ECMO patients (23).

(A) Anticoagulation should be initiated on postoperative day 1 with heparin with a goal-activated partial thromboplastin time (APTT) of 60–80 s. (B) During the whole process, APTT should not be used as the only indicator to guide anticoagulation. To adjust the dose of heparin, multiple indicators, such as platelet, D-II polymer, and fibrinogen, should be combined to comprehensively evaluate the coagulation state in patients. (C) Platelet consumption is tolerated with no bleeding and a platelet count of greater than 50,000 (B/L). (D) If heparin-induced thrombocytopenia is suspected, fondaparinux sodium or argatroban was used to replace heparin.

**V. Screening for lower extremity vascular ultrasound** In addition to a routine deep venous thrombus (DVT) risk screening, we performed point-of-care ultrasound DVT (POCUS DVT) examination and jugular vein examination on admission and every 7 days during ECMO for facilitating rapid diagnosis and treatment of DVT (24).

**FIGURE 1:**
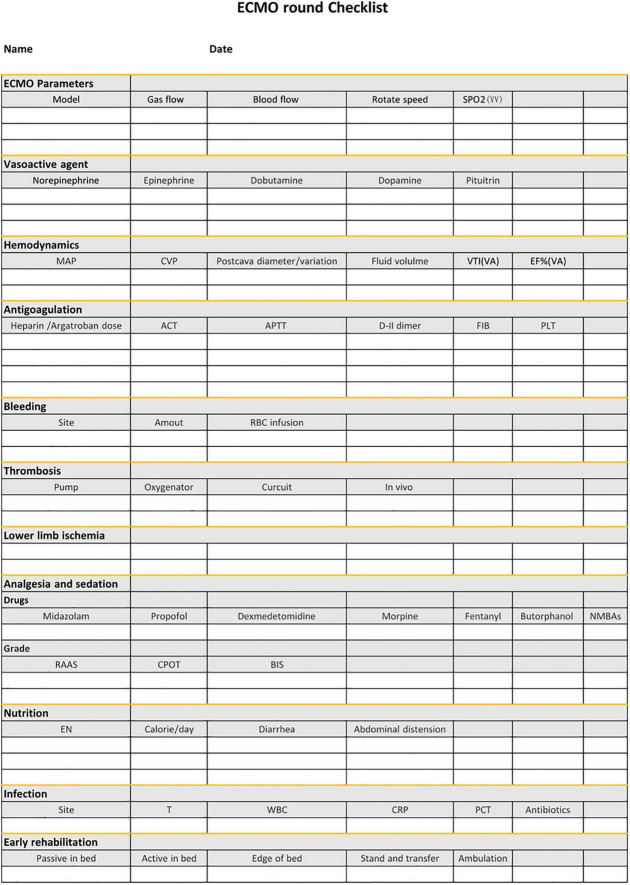
The checklist during the ward round following the intervention bundle. ECMO, extracorporeal membrane oxygenation; MAP, mean arterial pressure; CVP, central venous pressure; VTI, velocity time integral; EF%, ejection fraction; ACT, activated clotting time; APTT, activated partial thromboplastin time; FIB, fibrinogen; EN, enteral nutrition; T, temperature; WBC, white blood cells; CRP, C-reactive protein; PCT, procalcitonin.

**VI. Checklist** We established a shifting checklist including the intervention bundle to remind staff to manage patients elaborately and act the intervention program strictly (Figure 1).

**Interventions in the third stage** (August 2020–September 2020)—the rehabilitation with extracorporeal membrane oxygenation:

All ECMO recipients are evaluated daily from Monday to Saturday for their suitability for participation in physical and occupational therapy. The patients will be deferred therapy with the following situation: clinically significant hemorrhage, unstable arrhythmia, severe thrombocytopenia, hemodynamic instability requiring high-dose vasopressors, severe hypoxemia despite oxygen supplementation, sedation precluding active participation by the patient, and use of neuromuscular blockade (25, 26).

The rehabilitation regimen was completed by a team of two physical therapists (27). These sessions began initially with strengthening and reconditioning exercises in the supine position. As the patient’s tolerance improved, activity was quickly advanced to exercises in the sitting position on the edge of the bed to strengthen the upper and lower extremities. As strength increased, activities were advanced to cycling on the bed.

### Definition of complications

The following complications were recorded: (1) Bleeding events were defined according to the Extra-Corporeal Life Support Organisation (ELSO) definition (28): we defined a bleeding event if there was clinically overt bleeding recorded in the medical and/or nursing charts associated with either administration of 2 or more RBC units in 24 h or a drop in hemoglobin greater than 2 g/L over 24 h, or if there was a hemothorax, central nervous system or retroperitoneal bleeding, or if bleeding required an intervention. (2) Bloodstream infection: defined as occurring 24 h after the start of ECMO and 72 h after the withdrawal of ECMO (29). (3) Acute renal injury (AKI) was diagnosed according to RIFLE (30). (4) Thrombus includes clots in the oxygenator and other parts of the circuit and *in vivo* (31). (5) Lower limb ischemia refers to decreased or disappeared pulsation of the dorsal foot artery, pallor, and compartment syndrome or gangrene that may occur in severe cases (32). (6) Neurologic complication: defined as a cerebral hemorrhage or ischemia reported on a CT scan with no other potential etiology (33).

### Statistical analysis

Before analysis, all continuous variables were tested by the Kolmogorov-Smirnov test for normal distribution. Normally distributed variables are presented as mean ± SD, and non-normally distributed are presented as median values (interquartile range, IQR). Categorical variables are detailed as counts and/or percentages. The Mann-Whitney *U*-test was applied for non-parametric analyses of continuous variables. Analysis of categorical variables was performed using the Fisher exact test. Statistical regression models were constructed using multivariable logistic regression. A two-tailed test with a *P*-value less than 0.05 was considered statistically significant. A statistical package for the Social Science (SPSS Version 26.0; SPSS, Chicago, IL, USA) was used to perform all analyses.

A cumulative sum chart (CUSUM chart) was used to investigate a possible learning curve. We used the non-risk-adjusted cumulative observed minus expected failure graph procedure, such as that described by Banjas et al. (34). The CUSUM curve is calculated on the basis of the following considerations: where the ECMO treatment is successful, Xi = 0; and where the treatment is not successful, and the patient dies over the period of the hospital stay, Xi = 1. On the basis of epidemiological data from Karagiannidis et al. (35), we selected an acceptable error rate for ECMO treatment in Germany, which corresponds to the expected mortality of p0 = 0.6 for a mixed vv and va-ECMO population. The formula for calculation of the CUSUM curve was calculated as follows: Ci = C_(i–1)_ + (Xi–p0) with C_0_ = 0. The interpretation of the curve characteristics is carried out as follows: each successful treatment lowers the CUSUM value by p_0_, and the curve drops. Each failed treatment results in an increase of 1 - p_0_, and the curve rises. Where the actual error rate corresponds to the rate expected, the curve will oscillate along a horizontal axis.

## Results

### Baseline clinical characteristics

During the study period, a total of 297 ECMO-treated patients were evaluated. There were 70 patients included at baseline, and 227 patients were observed prospectively since the implementation of the intervention bundle. The baseline clinical characteristics of the patients separated according to the implementation period of the intervention bundle are shown in Table 1. Age, sex, and comorbidities were similar among the five periods. Most patients were supported with VA ECMO (68.7%) with a median (25th–75th percentile) duration in ECMO of 9.0 (4.0–15.0) days and a mean (SD) APECHII score 24.1 (7.5), in which there was no difference among all the groups. SAVE score and RESP score were similar among the five groups. The most frequent reason for ECMO treatment was myocardial infarction, followed by pneumonia for all periods. However, the proportion of extracorpareal cardiopulmonary resuscitation (ECPR) got higher and higher over time (*p* < 0.001). There were no differences in pre-ECMO lactate and the incidence of continuous renal replacement therapy (CRRT) during ECMO (Table 1).

**TABLE 1:** Baseline characteristics of the patients with extracorporeal membrane oxygenation (ECMO).

Variables	Baseline (*n* = 70)	During implementation (*n* = 75)	Post-implementation			*P*
			
			0–6 months (*n* = 45)	7–12 months (*n* = 52)	13–18 months (*n* = 55)	
Age, mean (SD)	53.8 (17.3)	56.2 (15.7)	58.9 (13.4)	59.0 (12.1)	56.2 (13.4)	0.226
Male, *n*(%)	50 (71.4)	50 (66.7)	30 (66.7)	30 (57.7)	36 (65.5)	0.640
BMI, kg/m^2^, mean (SD)	24.6 (4.8)	24.5 (4.1)	25.0 (4.1)	24.8 (4.4)	24.7 (2.9)	0.973
APECHII, mean (SD)	23.8 (7.3)	26.0 (7.7)	23.2 (6.3)	23.8 (8.3)	23.1 (7.1)	0.155
SAVE score, meidan (Q1-Q3)	−9.0 (−15.0 to −2.0)	−9.0 (−15.0 to −0.0)	−5.0 (−8.0 to −0.0)	−6.0 (−12.5 to −0.75)	−6.0 (−14.3 to −0.3)	0.555
RESP score, median (Q1-Q3)	2.0 (−1.0 to −3.0)	2.0 (−0.8 to −4.0)	1.5 (−0.8 to −3.0)	0.0 (−1.5 to −2.5)	0.0 (−2.0 to −2.0)	0.419
**Comorbidities, *n*(%)**						
Hypertension	25 (35.7)	22 (29.3)	14 (31.1)	21 (40.4)	22 (40.0)	0.620
DM	21 (30.0)	18 (24.0)	11 (24.4)	12 (23.1)	16 (29.1)	0.865
CKD	4 (5.7)	2 (2.7)	2 (4.4)	3 (5.8)	3 (5.5)	0.901
Indications, *n*(%)						0.874
Ischemic heart disease	31 (44.3)	38 (50.7)	28 (62.2)	24 (46.2)	28 (50.9)	
Myocarditis	7 (10.0)	8 (10.7)	3 (6.7)	4 (7.7)	6 (10.9)	
Pulmonary embolism	3 (4.3)	3 (4.0)	0 (0.0)	2 (3.8)	3 (5.5)	
Pneumonia	19 (27.1)	17 (22.7)	12 (26.7)	15 (28.8)	13 (23.6)	
IPF	6 (8.6)	2 (2.7)	2 (4.4)	3 (5.8)	2 (3.6)	
Others	4 (5.7)	7 (9.3)	0 (0)	4 (7.7)	3 (5.5)	
VA, *n*(%)	45 (64.2)	55 (73.3)	31 (68.8)	34 (65.3)	39 (70.9%)	0.777
ECRP, *n*(%)	5 (7.1)	10 (13.3)	10 (22.2)	18 (34.6)	18 (32.7)	< 0.001
Days on ECMO^a^	8.0 (2.0 to 15.3)	7.0 (3.0 to 14.0)	10.0 (5.0 to 16.5)	11.0 (6.0 to 17.0)	9.0 (5.0 to 13.0)	0.200
LOS in ICU^a^ (days)	15.0 (3.0 to 26.5)	13.0 (3.0 to 20.0)	15.0 (7.3 to 22.8)	15.5 (9.3 to 27.3)	14.0 (6.0 to 23.0)	0.808
Lac-preECMO (mmol/l)	6.0 (2.2 to 8.6)	6.0 (2.1 to 12.0)	3.9 (1.9 to 7.4)	4.3 (2.4 to 7.4)	5.5 (2.4 to 8.0)	0.369
CRRT, n(%)	25 (35.7)	28 (37.3)	18 (40.0)	21 (40.4)	18 (32.7)	0.857

ECMO, extracorporeal membrane oxygenation; BMI, body mass index; APECHII, acute physiology and chronic health evaluation II; SAVE, Survival after VA-ECMO; RESP, respiratory ECMO survival prediction; DM, Diabetes mellitus; CKD, chronic kidney disease; IPF, Idiopathic pulmonary fibrosis; VA, veno-Arterial; ECPR, Extracorporeal cardiopulmonary resuscitation; LOS, length of stay; CRRT, Continuous renal replacement therapy.

^*a*^Median (quartile).

### The compliance with the intervention bundle

To ensure the implementation of the bundle, compliance with the bundle was checked. We audited several aspects of the intervention limited by the resources. The compliance was the lowest for the use of the checklist, which was only 40.3% during the first 6 months after implementation, because of the increased burden. The usage increased to 87.5% after simplifying the form and strengthening education at 13–18 months. The compliance of perfusion cannulation and lung protection strategy reached up to almost 100% since the 7–12 months after the implementation. Overall, the compliance rates of the insertion bundle increased step by step (Table 2).

**TABLE 2:** Compliance of the strategies during post-implementation periods.

Strategies	0–6 months	7–12 months	12–18 months
Lung protection MV (%)	94.3	98.7	100.0
Early EN (%)	82.3	87.7	92.4
Early rehabilitation (%)	31.6	56.4	89.2
Time of operation (min)	18.6	15.4	10.7
Perfusion cannulation (%)	94.7	100.0	100.0
Checklist (%)	40.3	64.2	87.5

MV, mechanical ventilation; EN, enteral nutrition.

### Complications following extracorporeal membrane oxygenation

Among the 297 ECMO patients, 197 patients developed at least one complication. The two most common complications were bleeding and AKI during the whole study period, with the incidence of 36.0 and 31.6%, respectively. The incidence of bleeding and bloodstream infection decreased significantly, from 41.4 to 18.6% at baseline to 23.6 and 5.5% at 13–18 months after the implementation (*p* < 0.05). The incidence of neurologic complication and lower limb ischemia tended to decrease, which was the opposite trend in terms of thrombosis (Table 3).

**TABLE 3:** Incidence of complications during extracorporeal membrane oxygenation (ECMO) from baseline to 18 months of follow-up.

Period	Bleeding *n*(%)	AKI *n*(%)	Bloodstream infection *n*(%)	Lower limb ischemia *n*(%)	Thrombus *n*(%)	Neurologic complication *n*(%)
Baseline	29 (41.4)	22 (31.4)	13 (18.6)	6 (8.6)	15 (21.5)	4 (5.8)
During implementation	29 (38.6)	25 (33.3)	4 (5.4)	4 (5.4)	20 (16.6)	4 (5.4)
Post implementation						
0–6 months	18 (40.0)	13 (28.8)	3 (6.7)	6 (13.3)	16 (35.6)	1 (2.3)
7–12 months	18 (34.6)	19 (36.5)	5 (9.7)	4 (7.7)	18 (34.6)	4 (7.7)
13–18 months	13 (23.6)*	15 (27.2)	3 (5.5)*	1 (1.8)	14 (25.5)	2 (3.7)

ECMO, extracorporeal membrane oxygenation; AKI, acute kidney disease. *p < 0.05 compared with baseline period.

### Clinical outcomes

The mortality of ECMO patients was 57.1% at the baseline, which declined to 38.7% during the implementation period and further to 21.8% in the 13–18 months after the implementation (*p* < 0.05). Even compared with the during-period, mortality decreased significantly in the 13–18 months after the implementation (*p* < 0.05) (Table 4).

**TABLE 4:** Mortality of all extracorporeal membrane oxygenation (ECMO) patients and survival of extracorpareal cardiopulmonary resuscitation (ECPR) from baseline to 18 months of follow-up.

Outcome	Baseline	During implementation	Post implementation		
			
			0–6 months	7–12 months	13–18 months
Mortality n(%)	40 (57.1)	29 (38.7)*	15 (33.3)*	20 (38.5)*	12 (21.8)*^#^
Survival of ECPR n(%)	2 (40.0)	3 (30.0)	4 (40.0)	8 (44.4)	9 (50.0)

ECMO, extracorporeal membrane oxygenation; ECPR, Extracorporeal cardiopulmonary resuscitation. *p < 0.05 compared with baseline period. ^#^*p* < 0.05 compared with during implementation period.

**FIGURE 2:**
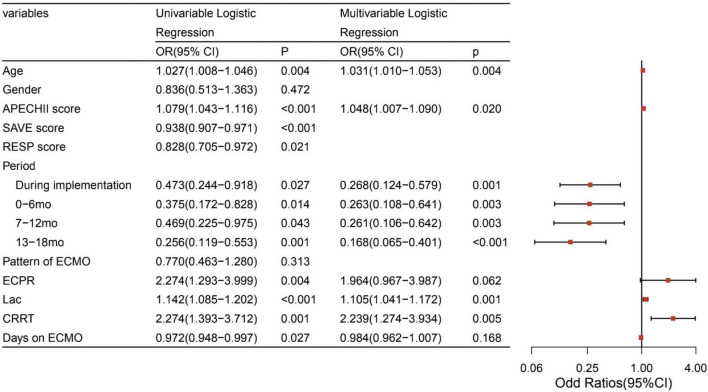
Factors associated with mortality of ECMO. ECMO, extracorporeal membrane oxygenation; APECHII, acute physiology and chronic health evaluation; SAVE, Survival after VA-ECMO; RESP, respiratory ECMO survival prediction; mo, month; ECPR, Extracorporeal cardiopulmonary resuscitation; Lac, lactate; CRRT, Continuous renal replacement therapy; LOS, length of stay.

**FIGURE 3:**
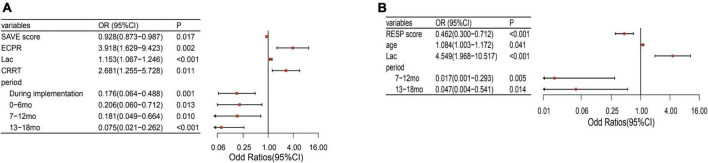
Factors associated with mortality of V-A and V-V ECMO separately. **(A)** Factors associated with mortality of V-A ECMO. **(B)** Factors associated with mortality of V-V ECMO. V-A, venoarterial; V-V, venovenous; ECMO, extracorporeal membrane oxygenation; SAVE, Survival after VA-ECMO; RESP, respiratory ECMO survival prediction; Lac, lactate; mo, month; CRRT, Continuous renal replacement therapy.

After adjusting for potential confounding factors, the intervention periods were still significantly associated with lower mortality (*P* < 0.05). The confounding factors that were independently associated with mortality were age, APECHII score, pre-ECMO lactate, and the CRRT during ECMO (Figure 2). Because of different underlying pathologies and different pathophysiology, there are different risk factors between these two modalities. Multilogistic regression was then carried out in V-V and V-A patients separately. It turned out that whether in V-V or V-A ECMO, this intervention bundle can effectively reduce mortality and improve the prognosis (Figure 3). But in V-V ECMO, the intervention bundle did not significantly reduce mortality until 7–13 months after the implementation.

Although the implementation of the bundle decreased ECMO mortality and the incidence of complications, there was no significant correlation between the incidence of complications and mortality in our study (Supplementary Table 1).

**FIGURE 4:**
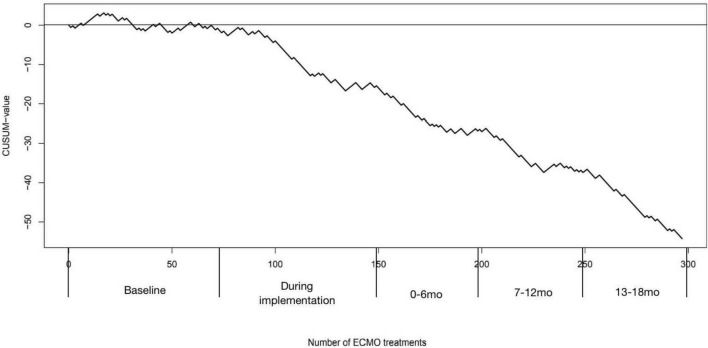
Cumulative observed minus failure (CUSUM) chart for in-hospital mortality after extracorporeal membrane oxygenation (ECMO) treatment. The vertical axis shows the CUSUM value, which increases for each failure or decreases on each success. The horizontal axis shows the number of ECMO treatments and the corresponding period. An upward slope represents a failure rate higher than expected and a downward slope represents a failure rate fewer than expected.

Figure 4 shows our CUSUM learning curve. The mortality rate initially increased followed by a horizontal progression, relating to a mortality rate that corresponds with the expected mortality rate *p*_0_ = 0.6, with a falling curve after the implementation of the intervention bundle, indicating the learning effect of a reduced mortality rate.

## Discussion

Within the last decade, ECMO, this complex, life-sustaining treatment mortality has experienced exponential growth in the world. Although ECMO can improve the survival of patients with advanced lung and heart disease, there is significant associated morbidity with the performance of this intervention (36). In our study, an intervention bundle established according to the literature and experience of our center reduced the mortality of ECMO. The effect increased gradually over the 18 months study period.

At baseline, the mortality of ECMO was even higher than the international level (37). However, during the implementation and the 18-month follow-up period, the mortality of ECMO decreased from 57.1 to 21.8% significantly, even with an increased proportion of ECPR. Moreover, in multivariable analysis, even after excluding the confounding factors, such as age, APECHII score, pre-ECMO lactate, and incidence of CRRT during ECMO, the intervention bundle was still significantly associated with lower mortality.

The survival of ECPR increased gradually throughout the study, although without significant difference. During the 13–18 months after bundle implementation, the survival of ECPR has reached up to 70%, which was much higher than in previous studies (38). This may be largely due to the refined management of hemodynamic management and accurate bedside ultrasound evaluation.

Although some studies have declared that medical complications associated with ECMO have a small impact on overall mortality, accounting only for 7% of fatal outcome cases (39). Emphasis must be put on experienced management of ECMO to further minimize their occurrence and impact on patient outcomes.

Bloodstream infection can potentially be a lethal complication. Bloodstream infection affected 11% of patients in ECMO in total in the study, which was in accordance with the literature reported where the percentage of patients who experienced bloodstream infections varied between 3.4 and 11.4% (40). The incidence of bloodstream infection showed a downward trend in general, but it rebounded at 7–12 months after implementation. Through the analysis of the quality control meeting and the playback of the surveillance video, we found that the incidence of AKI and usage of CRRT in this period was higher. The CRRT circuit was connected to ECMO. However, the aseptic operation at the junction was inadequate. Through our education and supervision, the incidence of bloodstream infection decreased significantly at 13–18 months, although the usage of CRRT did not change significantly. Surveillance with communication of the results to the ICU caregivers undoubtedly raises awareness of the magnitude of the problem and improves the medical quality.

For both types of ECMO, bleeding was the most common complication, which is in accordance with other literature (41–43). Compared with the baseline period, the incidence of bleeding decreased significantly at 13–18 months after bundle implantation, which may be due to a more reasonable evaluation of coagulation during ECMO. Although the incidence of bleeding was reduced, the incidence of thrombosis remained high. One of the reasons for this phenomenon is the point-of-care ultrasound DVT (POCUS DVT) examination was done sparingly in the baseline, which has become a routine operation later. Actually, coagulation during ECMO is still a global concern, and there is still no precise program to guide anticoagulation, which may be the focus of ECMO experts in future research.

The whole intervention was divided into three phases. In the early stage, due to the lack of operational experience, we focused on strengthening the operational and theoretical training of medical staff. ECMO can just provide an opportunity for the treatment of the primary disease, so only by focusing on the comprehensive management of the primary critical illness can we improve outcomes eventually. Therefore, in the second stage, we strengthened the management of hemodynamic and respiratory mechanics of patients. After the basic management of critically ill diseases is guaranteed, we strengthened the early rehabilitation exercise of ECMO through cooperation with rehabilitation medicine. The whole intervention was implemented step by step accompanied by the development of the ECMO center. Therefore, we believe that this study is meaningful for the construction of ECMO centers.

### Limitation

The study had several limitations. First, we could not evaluate the relative importance of individual components of the multifaceted interventions. However, our goal was a maximal improvement of patient’s outcome, and the present bundle offered a great probability of reducing mortality. Second, the sample size of the study was small and the observation period of the effect was short. There was only a trend of benefit but not a significant difference in several aspects. We may get more satisfactory results during a long-term observation or in a more large sample. Third, we did not analyze the results Last, as our province was still in the early stage of the construction of ECMO centers in 2019, we implemented the bundle that included almost all aspects of ECMO care and not focused on a specific target. However, the application of ECMO has now become increasingly widespread and quality in our province and even throughout China. Based on this, we will focus on specific problems that have been troubling us, such as anticoagulation during ECMO in the future. However, the present study still makes sense for the ECMO centers in the early stage.

## Conclusion

In summary, mortality of patients with ECMO can be effectively reduced by the intervention bundle and the benefit from the intervention was maintained throughout the 18-month study period.

## Data availability statement

The raw data supporting the conclusions of this article will be made available by the authors, without undue reservation.

## Ethics statement

The studies involving human participants were reviewed and approved by Qingdao University Affiliated Hospital Human Research Ethics Committee (QYFYKYLL931311921). Written informed consent for participation was not required for this study in accordance with the national legislation and the institutional requirements.

## Author contributions

JX contributed to the design of this study, interpretation of data, and writing of the manuscript. YJ analyzed the data and wrote the final manuscript. ZY, WZ, and XH conceived and designed the study. KS analyzed the data. QQ collected the data. All authors read and approved the final manuscript.
