# Supplementary material for: A phased intervention bundle to decrease the mortality of patients with extracorporeal membrane oxygenation in intensive care unit
Source: Front Med (Lausanne). 2022 Oct 17;9:1005162. doi: 10.3389/fmed.2022.1005162 (PMC9618597; doi:10.3389/fmed.2022.1005162)
Supplement: Supplementary file 1 [file Table_1.pdf]

Supplementary Table1 The association of the complication and mortality of ECMO

variables	Univariable Logistic Regression	
	OR(95% CI)	P
Bleeding	1.036(0.637-1.683)	0.888
AKI	1.427(0.868-2.348)	0.161
Infection	1.543(0.715-3.330)	0.269
Ischemia	0.696(0.327-1.482)	0.348
Thrombosis	0.859(0.508-1.452)	0.570
Neurologic complication	1.626(0.593-4.461)	0.345

ECMO=extracorporeal membrane oxygenation;AKI=acute kidney disease.
